# Burden and predictors of *Staphylococcus aureus* and *S. pseudintermedius* infections among dogs presented at an academic veterinary hospital in South Africa (2007–2012)

**DOI:** 10.7717/peerj.3198

**Published:** 2017-04-13

**Authors:** Daniel N. Qekwana, James Wabwire Oguttu, Fortune Sithole, Agricola Odoi

**Affiliations:** 1 Department of Paraclinical Sciences, Faculty of Veterinary Science, Section Veterinary Public Health, University of Pretoria, Pretoria, Gauteng, South Africa; 2 Department of Agriculture and Animal Health, College of Agriculture and Environmental Sciences, University of South Africa, Johannesburg, South Africa; 3 Ross University School of Veterinary Medicine, Basseterre, St Kitts and Nevis; 4 Biomedical and Diagnostic Sciences, College of Veterinary Medicine, The University of Tennessee, Knoxville, TN, United States of America

**Keywords:** *Staphylococcus*, *S. pseudintermedius*, *S. aureus*, Dogs, Canine, Predictors, Veterinary hospital, South africa, Multinomial logistic regression, Risk factors

## Abstract

**Background:**

Staphylococci are commensals of the mucosal surface and skin of humans and animals, but have been implicated in infections such as otitis externa, pyoderma, urinary tract infections and post-surgical complications. Laboratory records provide useful information to help investigate these infections. Therefore, the objective of this study was to investigate the burdens of these infections and use multinomial regression to examine the associations between various *Staphylococcus* infections and demographic and temporal factors among dogs admitted to an academic veterinary hospital in South Africa.

**Methods:**

Records of 1,497 clinical canine samples submitted to the bacteriology laboratory at a veterinary academic hospital between 2007 and 2012 were included in this study. Proportions of staphylococcal positive samples were calculated, and a multinomial logistic regression model was used to identify predictors of staphylococcal infections.

**Results:**

Twenty-seven percent of the samples tested positive for *Staphylococcus* spp. The species of *Staphylococcus* identified were * S. pseudintermedius* (19.0%), * S. aureus* (3.8%), * S. epidermidis* (0.7%) and *S. felis* (0.1%). The remaining 2.87% consisted of unspeciated *Staphylococcus*. Distribution of the species by age of dog showed that *S. pseudintermedius* was the most common (25.6%) in dogs aged 2–4 years while *S. aureus* was most frequent (6.3%) in dogs aged 5–6 years. * S. pseudintermedius* (34.1%) and *S. aureus* (35.1%) were the most frequently isolated species from skin samples. The results of the multivariable multinomial logistic regression model identified specimen, year and age of the dog as significant predictors of the risk of infection with *Staphylococcus*. There was a significant temporal increase (RRR = 1.17; 95% CI [1.06–1.29]) in the likelihood of a dog testing positive for * S. pseudintermedius* compared to testing negative. Dogs ≤ 8 years of age were significantly more likely to test positive for *S. aureus* than those >8 years of age. Similarly, dogs between 2–8 years of age were significantly more likely to test positive for *S. pseudintermedius* than those >8 years of age. In addition, dogs 2–4 years of age (RRR = 1.83; 1.09–3.06) were significantly more likely to test positive for *S. pseudintermedius* compared to those <2 years of age. The risk of infection with *S. pseudintermedius* or * S. aureus* was significantly higher in ear canal and skin specimens compared to other specimens.

**Conclusions:**

The findings suggest that *S. pseudintermedius* and *S. aureus* were the most commonly isolated species from dogs presented at the study hospital. Age of the dog and the location of infection were significant predictors of infection with both *Staphylococcus* species investigated. Significant increasing temporal trend was observed for * S. pseudintermedius* but not * S. aureus*. This information is useful for guiding clinical decisions as well as future research.

## Introduction

*Staphylococcus* bacteria are commensal organisms of mucosal surfaces and skin of both humans and animals but are also associated with a variety of diseases. The organisms can survive for months on environmental surfaces and serve as sources of infection (Neely & Maley, 2000; Coughenour, Stevens & Stetzenbach, 2011). Transmission from dogs to humans occurs following exposure to carrier or infected dogs (Pantosti, 2012; Faires, Tater & Weese, 2009; Frank et al., 2009; Boost, O’Donoghue & Siu, 2007). Due to this risk, some authors have recommended that, where possible, contact between animals and humans should be restricted to mitigate the risk of infection and its associated public health concerns (Faires, Tater & Weese, 2009).

*Staphylococcus* spp. in dogs have been isolated in several clinical conditions including: pyoderma, otitis, wound infections, sepsis, nasal infections, pneumonia, nephritis, and post-surgical infections (Weese & Van Duijkeren, 2010; Kawakami et al., 2010; Cohn & Middleton, 2010; Ishihara et al., 2010; Kramer, Schwebke & Kampf, 2006; May et al., 2005; Frank et al., 2003). However, the majority of infections have been associated with canine dermatologic conditions.

Due to the high rates of colonization of dogs with *Staphylococcus pseudintermedius* and *Staphylococcus aureus*, these species make up the majority of *Staphylococcus* related infections in dogs. For example, studies conducted in Canada, Hong Kong and Japan have reported the prevalence of *S*. *pseudintermedius* in dogs as ranging between 61% and 89.5% (Kawakami et al., 2010; Boost, O’Donoghue & Siu, 2007) whereas the prevalence of *S. aureus* ranges from 9% to 40% in dogs (Hanselman et al., 2009; Boost, O’Donoghue & Siu, 2007). Dogs also get infected with other species of *Staphylococcus* such as *S. schleiferi, S. epidermidis, S. xylosus* and *S. felis* (Chanchaithong et al., 2014).

There is paucity of information on the epidemiology of staphylococcal infections in dogs in South Africa. Specifically, there is no evidence of studies that have attempted to investigate temporal patterns of infections and the predictors of staphylococcal infections in dogs in South Africa. Furthermore, many of the investigations of predictors of infections described in the literature are based on the analysis of binary outcomes using logistic regression models, and there is no evidence of studies that have tried to ascertain if the predictors of the various *Staphylococcus* species differ using multinomial models. Therefore, the objective of this study was to investigate the burdens of these infections and use multinomial regression to examine the associations between infection with various *Staphylococcus* spp., and the demographic and temporal factors among dogs admitted to an academic veterinary hospital in South Africa.

## Materials and Methods

### Data collection and management

Data for this study were obtained from the bacteriology laboratory of the University of Pretoria academic veterinary hospital and included cases of *Staphylococcus* spp. infections isolated from all dog samples submitted to the laboratory for microbiological diagnosis between January 2007 and December 2012.

The data were assessed for duplicate entries. The dataset did not contain multiple tests from the same patient nor were there mixed infections in the samples analysed. *Staphylococcus* species were identified based on characteristics of the colony and chemical tests as described by Quinn et al. (1994). The laboratory records of all specimens from dogs submitted to the laboratory for diagnostic purposes were assessed. For the purpose of this study, only records of dogs from the Gauteng Province were included for analysis. This was to control for potential confounding by region as most of the samples were from Gauteng province and very few were from other provinces. Variables included in the dataset included age (in months), sex, breed, type of specimen submitted, address of the owner of the dog, and the date of specimen submission. The study excluded records of dogs with incomplete or inaccessible information (*n* = 12). The breed classification used in the study was adapted from the American Kennel Club (AKC), and included the following categories: working, sporting, herding, hound, toy, terrier, nonsporting and mixed breeds (American Kennel Club, 2016).

### Statistical analysis

#### Descriptive analysis

Age was categorised into five categories: <2 years, 2–4 years, 5–6 years, 7–8 years and >8 years. Crude and factor-specific proportions of *S. aureus* and *pseudintermedius* positive samples and their confidence intervals (95% CI) were computed. The factors (independent variables) included in these computations were breed, season, year, sex, age category and specimen type. In addition, annual changes in the proportion of samples that tested positive for *Staphylococcus* between 2007 and 2012 were displayed in temporal graphs.

#### Univariable and multivariable models

The first step in the investigation of the predictors of *Staphylococcus* infections in dogs was to fit univariable multinomial logistic regression models to assess the relationships between the potential predictors (sex, age, year, season, breed and specimen type) and the polytomous outcome variable, *Staphylococcus* status. This outcome variable represented the species of *Staphylococcus* (*S. pseudintermedius, S. aureus* and *Staphylococcus* negative that was used as the reference category). For this initial investigation, potential predictors at a *p*-value ≤ 0.20 were considered for inclusion in the multivariable model to be fitted in the second step. The variable ‘age’ was modelled as a categorical variable for ease of interpretation. Since the variable “specimen type” had too many categories to include in the model in its original form, it was re-coded into four categories (“ear canal”, urine, skin and “all others”) for inclusion in the model with “all others” as the referent category. To be able to assess temporal trend, the predictor variable “year” was included in the model as a continuous variable. However, since it does not have a meaningful interpretation at the value 0, it was scaled by subtracting the lowest value in the data (2007) before inclusion in the model.

In the second step, a multivariable multinomial logistic regression model was fit using manual backwards selection with the polytomous *Staphylococcus* status variable as the outcome. At this step, statistical significance was assessed at α = 0.05. Confounding was assessed by comparing the change in model coefficients with and without the suspected confounders. If the removal of a suspected confounding variable resulted in a 20% or greater change in another model coefficient, the removed variable was considered a confounder and retained in the model regardless of its statistical significance. All two-way interaction terms among variables in the final main effects model were also assessed. Additionally, linearity assumption of the predictor variable, year was assessed by examining the significance of adding a quadratic term to the model. The quadratic term was not statistically significant and hence it was removed from the model.

Relative risk ratios (RRRs) and their corresponding 95% confidence intervals were computed for all variables included in the final model. To directly assess if there were statistically significant differences between the predictors for *S. pseudintermedius* and *S. aureus,* the multinomial model was also fit with *S. pseudintermedius* as the reference category of the outcome (instead of the negative samples) and the predictors assessed for significance using an α = 0.05.

To assess the goodness-of-fit of the multinomial logistic regression models, ordinary logistic regression models were fit to each pairwise combination of the three potential outcome categories as proposed by Dohoo, Martin & Stryhn (2009). Hosmer-Lemeshow goodness-of-fit test was then used to assess these models. This approach was used because currently there are no available multinomial model fit assessment tests in SAS, the only software package available to the authors.

### Ethical statement

The study was approved (approval number: V051-14) by the Ethics Committee of the University of Pretoria.

## Results

### Characteristics of dogs tested

A total of 1,626 dogs were tested for *Staphylococcus* related infections between 2007 and 2012. Of these, 1,497 (92.1%) from Gauteng province were included in the study. The majority (86.4%) of dogs included in the study were from the City of Tshwane Municipality. Of these, 65.1% were from the Pretoria area (a suburb of Tshwane). The highest (24.7%) proportion of dogs was tested in 2009, and the lowest (7.8%) in 2012. Compared to other seasons, most samples (37.6%) were tested during summer months, while the lowest number was tested in spring (15.4%) (Table 1). The median age of the dogs in the study was 66 months (5 years) and the interquartile range was 27–101 months. Most dogs (29.9%) were >8 years old, followed by those <2 years (21.4%). The majority of samples tested were from female dogs (52.2%), with samples from males making up the remaining 47.8%.

**Table 1:** Temporal and Host-factor distribution of *Staphylococcus* infections among dogs presented at the academic veterinary hospital, 2007–2012.

Variable	^a^All samples tested		*S. pseudintermedius*		*S. aureus*	
	Proportion	^c^95% CI	^b^Proportion	^c^95% CI	^b^Proportion	^c^95% CI
**Year**						
2007	18.6 (279/1497)	16.8–20.7	14.3 (40/279)	10.7–18.9	2.9 (8/279)	1.5–5.6
2008	20.4 (305/1497)	18.4–22.5	19.3 (59/305	15.3–24.2	4.9 (15/305)	3.0–8.0
2009	24.7 (369/1497)	22.5–26.9	16.5 (61/369)	13.1–20.7	3.0 (11/369)	1.7–5.2
2010	15.6 (234/1497)	13.9–17.6	19.2 (45/234)	14.7–24.8	4.7 (11/234)	2.7–8.2
2011	12.9 (193/1497)	11.3–14.7	21.8 (42/193)	16.5–28.1	3.1 (6/193)	1.4–6.6
2012	7.8 (117/1497)	6.7–9.3	31.6 (37/117)	23.9–40.5	5.1 (6/117)	2.4–10.7
**Season**						
Autumn	20.0 (299/1497)	18.0–22.1	21.1 (63/299)	16.8–26.0	2 (6/299)	0.9–4.3
Spring	15.4 (230/1497)	13.6–17.2	21.3 (49/230)	16.5–27.0	4.3 (10/230)	2.4–7.8
Summer	37.6 (563/1497)	35.2–40.0	19.7 (111/563)	16.6–23.2	4.8 (27/563)	3.3–6.9
Winter	27.1 (405/1497)	24.9–29.4	15.1 (61/405)	11.9–18.9	3.5 (14/405)	2.1–5.7
**Age category**						
<2 years	21.4 (320/1497)	19.4–23.5	17.2 (55/320)	13.5–21.7	3.8 (12/320)	2.2–6.4
2–4 years	13.8 (207/1497)	12.2–15.7	25.6 (53/207)	21.0–33.0	5.3 (11/207)	3.0–9.3
5–6 years	16.8 (252/1497)	15.0–18.8	22.2 (56/252)	17.5–27.8	6.3 (16/252)	4.0–10.1
7–8 years	18.1 (271/1497)	16.2–20.1	23.6 (64/271)	19.0–29.0	4.4 (12/271)	2.6–7.6
>8 years	29.9 (447/1497)	27.6–32.2	12.5 (56/447)	9.8–15.9	1.3 (6/447)	0.6–2.9
**Sex**						
Female	52.2 (767/1468)	49.7–54.8	17.2 (132/767)	12.5–19.7	4.3 (33/767)	3.1–6.0
Male	47.8 (701/1468)	45.2–50.3	21.0 (147/701)	18.1–24.1	3.4 (24/767)	2.3–5.0
**Breed**						
Working	26.3 (387/1474)	24.01–28.6	16 (61/387)	12.5–19.7	3.9 (15/387)	2.4–6.3
Sporting	18.0 (265/1474)	16.1–20.0	22.3 (59/265)	17.7–27.7	3.4 (9/265)	1.8–6.3
Herding	12.0 (177/1474)	10.5–13.8	14.1 (25/177)	9.7–20.0	5.1 (9/177)	2.7–9.4
Hound	11.1 (163/1474)	9.6–12.8	14.1 (23/163)	9.6–20.3	4.3 (7/163)	2.1–8.6
Toy	11.1 (163/1474)	9.6–12.8	16.6 (27/163)	11.6–23.0	3.1 (5/163)	1.3–7.0
Terrier	10.7 (158/1474)	9.2–12.4	26.6 (42/158)	20.3–34.0	3.8 (6/158)	1.8–8.0
Nonsporting	6.5 (95/1474)	5.3–7.8	29.5 (28/95)	21.3–39.3	2.1 (2/95)	0.6–7.4
Crossbreed	4.8 (71/1474)	3.8–6.0	18.3 (13/71)	11.0–28.9	5.6 (4/71)	2.2–13.6
**Specimen**						
Urine	31.0 (463/1493)	28.7–33.4	4.5 (21/463)	3.0–6.8	1.7 (8/165)	2.5–9.3
Ear canal	17.0 (254/1493)	15.2–19.0	34.7 (88/254)	29.1–40.7	5.1 (13/254)	3.0–8.6
Skin	11.1 (165/1493)	9.6–12.7	58.8 (97/165)	51.2–66.0	12.1 (20/165)	8.0–18.0
All others	40.9 (611/1493)	38.5–43.4	12.8 (78/611)	10.4–15.7	2.6 (16/611)	1.6–4.2

**Notes.**

a All samples tested included those tested for all species of *Staphylococcus* (not just *S. pseudintermedius* and *S. aureus*).

b Proportion of positive samples under each category.

c 95% confidence interval.

A total of seventy-three breeds of dogs were identified, and of these 11.8% were Boerboel followed by German Shepherd (9.1%), Bullterrier (7.0%), Dachshund (6.7%), Labrador Retriever (5.0%), Jack Russel (4.8%), Crossbreed (4.7%), Rottweiler (3.3%), Spaniel (2.8%), Yorkshire Terrier (2.8%), Maltese (2.5%), English Bulldog (2.4%), Husky (2.2%), Border Collie (2.1%) and Great Dane (2.1%). For purposes of this study, breeds that made up <2% of the study population were classified as “all others”, and all together this group contributed 30.8% of the samples submitted and processed. When the breeds were further classified according to the American Kennel Club (AKC) dog classification system, the highest proportion of samples tested were from working breeds (25.9%) while samples from crossbreeds (4.8%) made up the lowest proportion of submissions (Table 1).

A total of 48 different types of specimens were submitted and classified into four main groups: urine (31.0%), ear canal swabs (17.0%), skin (11.0%) and other specimen types classified as ‘all others’ (40.9%).

### Isolated Staphylococcus species

Of the 1,497 samples tested, 26.5% (396/1497) were positive for *Staphylococcus* spp. From the submitted diagnostic samples, *S. pseudintermedius* was isolated most often (19.0%, 284/1497), followed by *S. aureus* (3.8%, 57/1497), unspeciated *Staphylococcus* (2.9%, 43/1497), *S. epidermidis* (0.7%, 11/1497) and one *S. felis* (0.1%, 1/1497). The majority (96.7%, 341/353) of *Staphylococcus* spp. identified were coagulase-positive (*S. pseudintermedius* and *S. aureus*), and only 3.3% (12/353) were coagulase negative (*S. epidermidis* and *S. felis*).

**Figure 1:**
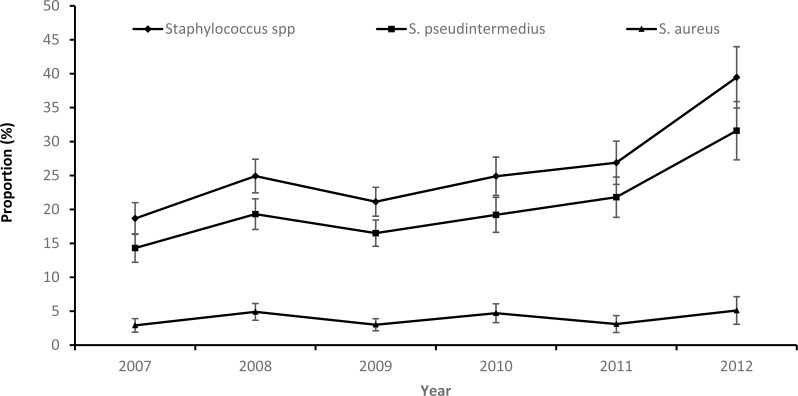
Annual patterns in the proportions and 95% confidence intervals of staphylococcus infections among canine samples tested at the academic veterinary hospital, 2007–2012.

While there was an overall increasing temporal trend in both the annual proportion of samples that tested positive for *Staphylococcus* spp. or *S. pseudintermedius* during the study period, the proportion of *S. aureus* positive samples remained stable (Fig. 1).

**Figure 2:**
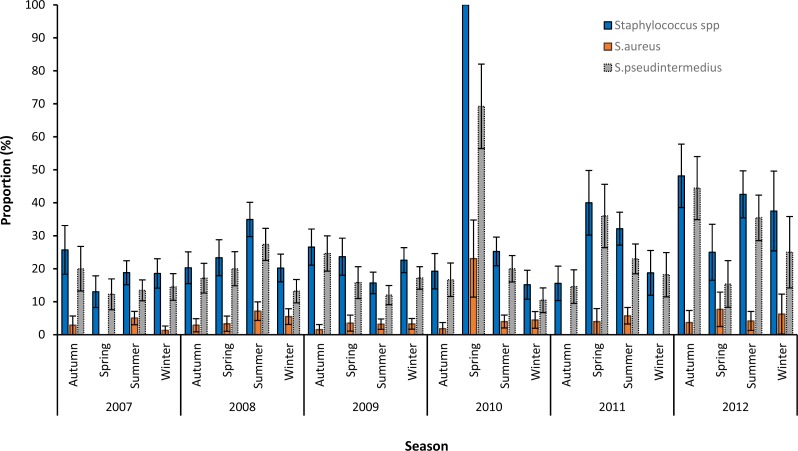
Seasonal patterns in the proportions and 95% confidence intervals of *Staphylococcus* infections among canine samples tested at the academic veterinary hospital, 2007–2012.

Based on graphing of the crude proportions of samples that tested positive for *Staphylococcus* spp., S. *aureus* and S. *pseudintermedius*, no seasonal patterns in the distribution of the three variables were apparent (Fig. 2). The very high proportion of *Staphylococcus* positive samples (100%) and *S. pseudintermedius* (69.2%) seen in spring of 2010 could be attributed to the small numbers of samples submitted during that season.

Overall, 2012 had the highest proportion of samples that were positive for *S. pseudintermedius* (31.6%) and *S. aureus* (5.1%). The highest proportion of *S. pseudintermedius* positive samples were in Autumn (21.1%) and Spring (21.3%), while the highest proportion of *S. aureus* positive samples were in Summer (4.8%) (Table 1).

*S. pseudintermedius* was most common in 2–4 year old dogs (25.6%) followed by dogs in the 7–8 year age category (23.6%). On the other hand, *S. aureus* was most common in dogs 5–6 years old (6.3%) followed by dogs 2–4 years old (5.3%). Male dogs had the highest proportion of samples that tested positive for *S. pseudintermedius* (21.0%), while females had the highest proportion of samples that tested positive for *S*. *aureus* (4.3%) (Table 1).

Overall, non-sporting breeds had the highest proportion (29.5%) of samples positive for *S. pseudintermedius* followed by terriers (26.6%). By contrast, crossbreeds had the highest proportion (5.6%) of samples that tested positive for *S. aureus* followed by herding breeds (5.1%) (Table 1).

**Figure 3:**
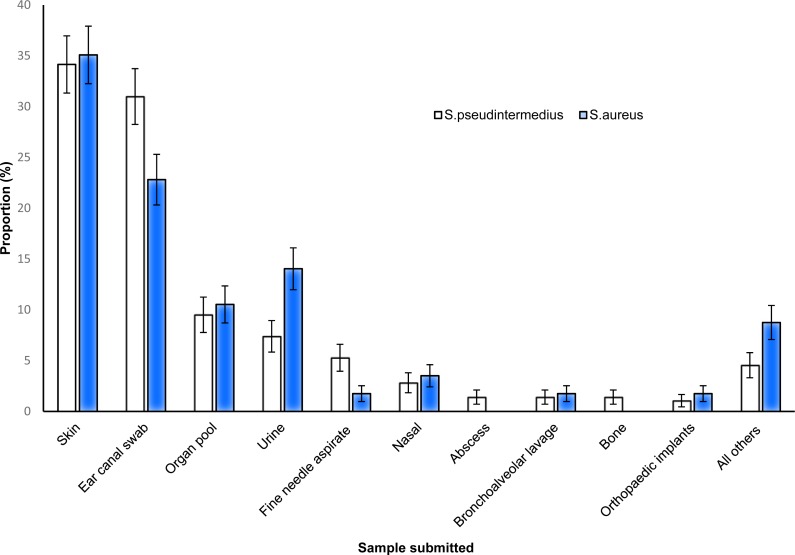
Distribution of the proportions and 95% confidence intervals of *S. pseudintermedius* and *S. aureus* infections among canine samples tested at the academic veterinary hospital, 2007–2012.

*S. pseudintermedius* and *S. aureus* were commonly isolated from both skin and ear samples, while abscesses and bone samples did not yield any *S. aureus* (Fig. 3).

### Predictors of staphylococcus infections based on the multinomial logistic regression

Following assessments using univariable models, age (*p* = 0.0003), season (*p* = 0.1435), breed (*p* = 0.0588), year (*p* = 0.0004), specimen type (*p* < 0.0001) and sex (*p* = 0.0728) were considered for inclusion in the multivariable model based on a liberal *p*-value of 0.20 (Table 2). However, in the multivariable multinomial model only age, specimen-type and year were significantly associated with infection classification (Table 3).

**Table 2:** Results of univariable multinomial logistic models investigating potential predictors of canine *Staphylococcus* infections in samples tested at the academic veterinary hospital, 2007–2012.

Variables	*S. aureus*				*S. pseudintermedius*			
	^a^RRR	^b^95% CI		*p*-values	^a^RRR	^b^95% CI		*p*-values
**Season**								
Autumn	0.60	0.23	1.58	0.2975	1.44	0.97	2.13	0.0696
Spring	1.37	0.60	3.16	0.4582	1.54	1.01	2.35	0.0441
Summer	1.46	0.75	2.83	0.2652	1.38	0.97	1.94	0.0710
Winter	Ref	.	.	.	.	.	.	.
**Breed**								
Crossbreed	1.50	0.48	4.70	0.4862	1.20	0.61	2.34	0.5943
Herding	1.28	0.54	2.99	0.5753	0.87	0.52	1.45	0.5953
Hound	1.04	0.41	2.61	0.9363	0.84	0.50	1.42	0.5099
Nonsporting	0.60	0.13	2.68	0.5016	2.06	1.22	3.47	0.0068
Sporting	0.92	0.40	2.15	0.8486	1.48	0.99	2.22	0.0544
Terrier	1.08	0.41	2.86	0.8723	1.86	1.19	2.93	0.0069
Toy	0.76	0.27	2.13	0.6015	1.01	0.61	1.66	0.9744
Working	Ref	.	.		.	.	.	.
**Sex**								
Female	1.20	0.70	2.06	0.5021	0.78	0.60	1.02	0.0728
Male	Ref	.	.		.	.	.	
**Age**								
<2 years	2.62	0.97	7.07	0.0577	1.29	0.86	1.93	0.2252
2–4 years	4.46	1.62	12.29	0.0039	2.30	1.50	3.53	0.0001
5–6 years	5.09	1.96	13.25	0.0009	1.91	1.26	2.89	0.0022
7–8 years	2.73	1.01	7.38	0.0477	1.56	1.05	2.32	0.0276
>8 years	Ref	.	.		.	.	.	.
**Year**								
2007–2012	1.08	0.907	1.285	0.3876	1.167	1.071	1.272	0.0004
**Specimen**								
Ear canal	2.83	1.33	6.02	0.01	3.93	2.75	5.61	<.0001
Urine	0.59	0.25	1.39	0.22	0.32	0.19	0.52	<.0001
Skin	14.70	7.09	30.48	<.0001	14.63	9.48	22.57	<.0001
All others	.	.	.		.	.	.	.

**Notes.**

a Relative Risk Ratio.

b 95% Confidence Intervals.

**Table 3:** Results of the final multinomial logistic model showing predictors of *Staphylococcus* species infections in dogs presented at the academic veterinary hospital, 2007 and 2012.

Variables	*S. aureus*				*S. pseudintermedius*			
	^a^RRR	^b^95% CI		*p*-value	^a^RRR	^b^95% CI		*p*-value
**Age**								
<2 years	2.93	1.04	8.22	0.0416	1.26	0.78	2.05	0.3406
2–4 years	4.27	1.49	12.27	0.007	2.31	1.39	3.84	0.0012
5–6 years	5.10	1.89	13.78	0.0013	2.06	1.27	3.36	0.0037
7–8 years	2.49	0.89	6.96	0.083	1.41	0.89	2.25	0.1479
>8 years	Ref	.	.	.	.	.	.	.
**Specimen**								
Ear canal	3.13	1.44	6.82	0.0041	3.99	2.72	5.84	<.0001
Urine	0.56	0.23	1.34	0.1901	0.32	0.19	0.53	<.0001
Skin	15.42	7.21	33.00	<.0001	14.26	9.02	22.55	<.0001
All others	Ref	.	.	.	.	.	.	.
**Time**								
Year (scaled)	1.09	0.91	1.30	0.3597	1.17	1.06	1.29	0.0027

**Notes.**

a Relative Risk Ratio.

b 95% Confidence Intervals.

Compared to dogs >8 years of age, the following age classes were significantly more likely to have dogs that tested positive for *S. aureus* compared to testing negative for staphylococci: <2 years, 2–4 years and 5–6 years (Table 3). Similarly, 2–4 and 5–6 year old dogs compared to >8 year old dogs, were significantly more likely to test positive for *S. pseudintermedius* than test negative. In addition, dogs 2–4 years of age (RRR = 1.83, 95% CI [1.09–3.06], *p* = 0.0221) compared to dogs <2 years of age were significantly more likely to test positive for *S. pseudintermedius* than testing negative (Table 4).

**Table 4:** Results of changing reference categories of categorical variables included in the final model presented in Table 3.

Variables	*S. aureus*				*S. pseudintermedius*			
	^a^RRR	^b^95% CI		*p*-value	^a^RRR	^b^95% CI		*p*-value
**Age**								
<2 years	1.18	0.5	2.78	0.7094	0.9	0.56	1.45	0.654
2–4 years	1.72	0.71	4.19	0.2334	1.64	0.99	2.71	0.0548
5–6 years	2.05	0.91	4.65	0.0852	1.46	0.9	2.37	0.1225
>8 years	0.4	0.14	1.13	0.083	0.71	0.45	1.13	0.1479
7–8 years	Ref	.	.	.	.	.	.	.
<2 years	0.57	0.25	1.31	0.1852	0.61	0.37	1.01	0.0538
2–4 years	0.84	0.36	1.96	0.6823	1.12	0.67	1.88	0.6658
7–8 years	0.49	0.22	1.11	0.0852	0.68	0.42	1.11	0.1225
>8 years	0.2	0.07	0.53	0.0013	0.49	0.3	0.79	0.0037
5–6 years	Ref	.	.	.	.	.	.	.
<2 years	0.69	0.28	1.67	0.4049	0.55	0.33	0.92	0.0221
5–6 years	1.19	0.51	2.79	0.6823	0.89	0.53	1.5	0.6658
7–8 years	0.58	0.24	1.42	0.2334	0.61	0.37	1.01	0.0548
>8 years	0.23	0.08	0.67	0.007	0.43	0.26	0.72	0.0012
2–4 years	Ref	.	.	.	.	.	.	.
2–4 years	1.46	0.6	3.55	0.4049	1.83	1.09	3.06	0.0221
5–6 years	1.74	0.77	3.96	0.1852	1.63	0.99	2.68	0.0538
7–8 years	0.85	0.36	2.01	0.7094	1.12	0.69	1.8	0.654
>8 years	0.34	0.12	0.96	0.0416	0.79	0.49	1.28	0.3406
<2 years	Ref	.	.	.	.	.	.	.
**Specimen**								
All others	0.32	0.15	0.7	0.0041	0.25	0.17	0.37	<.0001
Urine	0.18	0.07	0.45	0.0003	0.08	0.05	0.14	<.0001
Skin	4.93	2.18	11.12	0.0001	3.58	2.22	5.76	<.0001
Ear canal	Ref	.	.	.	.	.	.	.
All others	0.07	0.03	0.14	<.0001	0.07	0.04	0.11	<.0001
Urine	0.04	0.01	0.09	<.0001	0.02	0.01	0.04	<.0001
Ear canal	0.2	0.09	0.46	<.0001	0.28	0.17	0.45	<.0001
Skin	Ref	.	.	.	.	.	.	.

**Notes.**

a Relative Risk Ratios.

b 95% Confidence Intervals.

The type of specimen tested was also significantly associated with both *S. aureus* and *S. pseudintermedius.* Ear canal and skin specimens were more likely to test positive (as opposed to testing negative) for *S. pseudintermedius* and *S. aureus* compared to specimen categorized as ‘all others’. However, urine samples were less likely to test positive (as opposed to testing negative) for *S. pseudintermedius* compared to specimen classified as ‘all others’. In addition, there was a significant temporal increase (RRR = 1.17, 95% CI [1.06–1.29], *p* = 0.0027) in the likelihood of a dog testing positive to *S. pseudintermedius* compared to testing negative.

Compared to skin and ear canal samples, the other specimen types were significantly less likely to be infected with either *Staphylococcus* species (Table 4), and skin specimens were significantly more likely to be infected with either bacterial species than ear canal samples (Table 4).

To directly assess if there were statistically significant differences between the predictors of *S. pseudintermedius* and *S. aureus*, a multinomial model was fit with *S. pseudintermedius* positive as the reference group (instead of the negative samples). The results of this model showed no significant differences between the predictors of *S. pseudintermedius* and those of *S. aureus* (Table 5). Effects of changing the reference categories of the categorical predictors of the model presented in Table 5 is shown in Table 6.

**Table 5:** Results of the final multinomial logistic model showing predictors of *Staphylococcus* spp. infection with *S. pseudintermedius* as the reference category, 2007–2012.

	*Staphylococcus* Negative				*S. aureus*			
	^a^RRR	^b^95% CI		*p*-value	^a^RRR	^b^95% CI		*p*-value
**Age**								
<2 years	0.79	0.49	1.28	0.3406	2.31	0.79	6.79	0.1262
2–4 years	0.43	0.26	0.72	0.0012	1.85	0.63	5.47	0.2667
5–6 years	0.49	0.30	0.79	0.0037	2.47	0.88	6.94	0.0851
7–8 years	0.71	0.45	1.13	0.1479	1.76	0.61	5.11	0.2968
8>years								
**Specimen**								
Ear canal	0.25	0.17	0.37	<.0001	0.79	0.35	1.78	0.5613
Urine	3.14	1.88	5.22	<.0001	1.75	0.65	4.70	0.2695
Skin	0.07	0.04	0.11	<.0001	1.08	0.51	2.29	0.838
All others								
**Time**								
Year	0.86	0.78	0.95	0.0027	0.93	0.77	1.12	0.4608

**Notes.**

a Relative Risk Ratio.

b 95% Confidence Intervals.

**Table 6:** Results of changing reference categories of categorical variables included in the final model presented in Table 5.

	*Staphylococcus* Negative				*S. aureus*			
	^a^RRR	^b^95% CI		*p*-value	^a^RRR	^b^95% CI		*p*-value
**Age**								
2–4 years	0.55	0.33	0.92	0.0221	0.8	0.32	2.01	0.6335
5–6 years	0.61	0.37	1.01	0.0538	1.07	0.45	2.54	0.8796
7–8 years	0.9	0.56	1.45	0.654	0.76	0.31	1.87	0.5522
8>years	1.26	0.78	2.05	0.3406	0.43	0.15	1.27	0.1262
<2 years								
<2 years	1.83	1.09	3.06	0.0221	1.25	0.5	3.15	0.6335
5–6 years	1.12	0.67	1.88	0.6658	1.34	0.56	3.21	0.5144
7–8 years	1.64	0.99	2.71	0.0548	0.95	0.38	2.38	0.918
8>years	2.31	1.39	3.84	0.0012	0.54	0.18	1.6	0.2667
2–4 years								
<2 years	1.63	0.99	2.68	0.0538	0.94	0.39	2.22	0.8796
2–4 years	0.89	0.53	1.5	0.6658	0.75	0.31	1.79	0.5144
7–8 years	1.46	0.9	2.37	0.1225	0.71	0.3	1.67	0.4347
8>years	2.06	1.27	3.36	0.0037	0.4	0.14	1.13	0.0851
5–6 years								
<2 years	1.12	0.69	1.8	0.654	1.313	0.535	3.225	0.5522
2–4 years	0.61	0.37	1.01	0.0548	1.049	0.42	2.619	0.918
5–6 years	0.68	0.42	1.11	0.1225	1.404	0.599	3.289	0.4347
8>years	1.41	0.89	2.25	0.1479	0.567	0.196	1.645	0.2968
7–8 years								
**Specimen**								
Urine	12.51	7.37	21.22	<.0001	2.23	0.8	6.2	0.1257
Skin	0.28	0.17	0.45	<.0001	1.38	0.62	3.04	0.4279
All others	3.99	2.72	5.84	<.0001	1.27	0.56	2.89	0.56
Ear canal								
Ear canal	0.08	0.05	0.14	<.0001	0.45	0.16	1.25	0.1257
Skin	0.02	0.01	0.04	<.0001	0.62	0.23	1.64	0.3355
All others	0.32	0.19	0.53	<.0001	0.57	0.21	1.54	0.2695
Urine								
Ear canal	3.58	2.22	5.76	<.0001	0.73	0.33	1.6	0.4279
Urine	44.72	24.81	80.62	<.0001	1.62	0.61	4.29	0.3355
All others	14.26	9.02	22.55	<.0001	0.93	0.44	1.96	0.838
Skin								

**Notes.**

a Relative Risk Ratios.

b 95% Confidence Intervals.

## Discussion

This study was designed to assess the burdens and predictors of staphylococcal infections in dogs presented at an academic veterinary hospital in South Africa between 2007 and 2012. We used multinomial logistic regression models to investigate predictors of staphylococcal infections to assess if there were significant differences in predictors across species or if the strength of association differed. Therefore, the results of this study contribute significantly to improving our understanding of the epidemiology of staphylococcal bacterial infections in dogs presented at an academic veterinary hospital in South Africa.

Our results show that *Staphylococcus* bacteria were isolated from 26.5% of samples submitted to the academic veterinary hospital. This is lower than the 55.1% reported by Wedley et al. (2014) in mainland United Kingdom (UK) and 67.7% by Lilenbaum et al. (2000) in Brazil. The low proportion of *Staphylococcus* positive samples identified in this study compared to the above mentioned studies may be due to differences in target populations. In this study, we assessed staphylococcal infections among clinical cases of hospitalized dogs, whereas, Wedley et al. (2014) assessed infections in outpatient dogs and Lilenbaum et al. (2000) specifically investigated infection in dogs with otitis.

The significant increasing annual trend in the proportion of *Staphylococcus pseudintermedius* infections has not been previously reported/investigated in other dog populations. However, this could be attributed to increased access to veterinary services in South Africa since the dawn of democratic South Africa. This would especially be true if there is systematic access to and utilization of veterinary services such that dogs that potentially have high *Staphylococcus* infection risks are more likely to be presented for diagnostic services at the academic veterinary hospital compared to those with lower staphylococcal infection risks. It is also possible that the increase in the proportions of staphylococcal related infections in this study may be due to progressive poor health and wellbeing of dogs living in and around the area which makes them susceptible to staphylococcal infections. Furthermore, changes in referral practices by surrounding veterinary clinics, changes in owner attitudes towards seeking treatment for chronic skin conditions, and/or changes in lab procedures could have resulted in the increased identification of these infections. Evidence from the current study showed no seasonal pattern in the risk of canine *S. pseudintermedius* or *S. aureus* infection. This contrasts with reports of seasonal atopic dermatitis with secondary staphylococcal infections that has been reported by other authors (Simou et al., 2005).

Our study found that Coagulase-Positive *Staphylococcus* (CoPS) organisms were more common than Coagulase-Negative *Staphylococcus* (CoNS). This is consistent with the findings by Wedley et al. (2014) who reported higher proportions of CoPS (38%) in dogs compared to CoNS (19%) in the UK. In contrast, Schmidt et al. (2014), in a UK study and Lilenbaum et al. (2000) in a Brazilian study reported a higher frequency of CoNS (95% and 61%) than CoPS (41% and 39%) in dogs. However, it should be noted that in contrast to our study, Schmidt et al. (2014) tested samples collected from healthy Labrador Retrievers. These could explain the differences in the proportions of dogs with CoPS and CoNS infections in the two studies.

*S. pseudintermedius* was more commonly isolated (19.0%) than *S. aureus* in this study. However, the observed proportion was lower than 87% reported in Switzerland (Gandolfi-Decristophoris et al., 2013) and 69% reported in Denmark (Paul et al., 2012). Although the prevalence of *S. pseudintermedius* was higher than that of *S. aureus*, there was no significant difference between the two species in terms of their affinity for different sample types. Nonetheless, a higher proportion of *S. pseudintermedius* infections was observed from skin (34.1%) and ear (31.0%) samples compared to other sites/specimens. This is not unexpected because previous studies have reported higher frequency of *S. pseudintermedius* in dogs with pyoderma or otitis (Wedley et al., 2014; Griffeth et al., 2008; Lilenbaum et al., 2000). These findings can be explained by the fact that *S. pseudintermedius* is known to be more adapted to colonise the skin surface of dogs than *S. aureus* (Gandolfi-Decristophoris et al., 2013; Schmidt et al., 2014; Kawakami et al., 2010). Moreover, increased adherence by *S. pseudintermedius* to corneocytes of canines with atopic dermatitis has been previously reported (McEwan, Mellor & Kalna, 2006) suggesting that dogs are more likely to be infected with *S. pseudintermedius* compared to other species. Additionally, the results of the study show that ear canal and skin sites compared to other sites are more likely to test positive for *S. pseudintermedius* or *S. aureus* than test negative. This further supports research that reports that skin and ear canal compared to other body sites are more likely to be infected with *Staphylococcus* organism than other bacterial organisms (Gandolfi-Decristophoris et al., 2013; Schmidt et al., 2014; Kawakami et al., 2010).

The high frequency of *S. pseudintermedius* in dogs visiting the academic veterinary hospital is of public health significance because of association between *S. pseudintermedius* colonization in dogs and infection in humans with a history of exposure to carrier dogs (Hanselman et al., 2009). Since *S*. *pseudintermedius* is not a commensal in the nasal passages of healthy humans, dogs are thought to be an important reservoir for human infections with this pathogen (Tanner et al., 2000).

Although the results of the descriptive analysis indicated that female dogs carried more *S. aureus*, the results from the multinomial logistic regression indicated that sex of the dog was not a significant predictor of staphylococcal infections in dogs presented at the academic veterinary hospital in South Africa. This is consistent with the findings by Hanselman et al. (2009) who reported no significant association between sex and staphylococcal infections in dogs in Canada. However, our findings are in contrast to findings by Boost, O’Donoghue & James (2008), who reported that female dogs in Japan were more likely to be infected with *S. aureus* compared to their male counterparts.

The significant association between age and the risk of *Staphylococcus* infection in dogs observed in the present study was anticipated because age has previously been described as a predictor for *Staphylococcus* infection in dogs (Boost, O’Donoghue & James, 2008). However, unlike the study by Boost, O’Donoghue & James (2008) that reported more colonization in older dogs compared to puppies and middle aged dogs, in this study dogs ≤6 years of age and 2–6 years of age compared to dogs >8 years of age had a significantly higher risk of *S. aureus* and *S. pseudintermedius* infection, respectively, compared to testing negative. It was also noted that the risk of *S. pseudintermedius* infection was significantly higher in 2–4 year old dogs compared to dogs <2 years of age.

The external validity of this study should be interpreted with caution since it is based on submitted laboratory samples. Moreover, the history of previous use of antibiotics or anti-inflammatory agents was not included in the analysis. It is possible that this could have affected the recovery rates of *Staphylococcus* species. Since the population under study did not include outpatient cases, the study population should not be regarded as representative of the dog population in Gauteng or of those visiting the teaching hospital. Nonetheless, the results provide a useful preliminary indication of the burden and predictors of staphylococcal infections in dogs presented to the academic veterinary hospital.

## Conclusions

This study confirmed that skin, ear and urinary tract infections in dogs presented at the academic veterinary hospital are mainly due to *S. pseudintermedius* followed by S. *aureus*. Furthermore, the risk of infection with *S. aureus or S. pseudintermedius* in dogs presented at the academic veterinary hospital differed by age, with dogs ≤8 years of age often being at higher risk of infection compared to those >8 years old. This is useful information to guide clinical decisions and future studies.

## Additional Information and Declarations

### Data Availability

The following information was supplied regarding data availability: The data used in the study is owned by a third party (the bacteriology lab of the University Pretoria Veterinary Academic Hospital) who has not given their permission to publish the raw data as part of this manuscript.
